# Our Penal and Prison System.—I

**Published:** 1922-09

**Authors:** 


### OUR PENAL AND PRISON SYSTEM.—I.

INTEREST has always been taken in prisons, although it has varied in amount from time to time. And this interest should be stimulated and directed by the recent publication of two books on the subject. " English Prisons under Local Government," by Sidney and Beatrice Webb (Longmans, Green & Co., 15s. net), forms part of these authors' well-known study of English local government, the volumes of which are so justly appreciated by all students. It is furnished with a preface by Mr. Bernard Shaw, who writes with all his accustomed vigour and whose remarks on the subject of punishment are worthy of careful consideration, although the value of the book, as a scientific treatise, is to some extent spoiled by his exaggerated language. The second book to which we refer is entitled " English Prisons To-day " (same publishers, 25s. net). It is the report of a Prison System Inquiry Committee, and is edited by Messrs. Stephen Hobhouse and Fenner Brockway. An enormous amount of work has clearly been given to the production of both these volumes. They should be read together, for they are complementary. Their publication gives us an opportunity for a review of the whole subject. And we hope it may also cause some official investigation to be made, in order that our entire penal and prison systems may be reconsidered in the light of the fuller knowledge which we now possess. The prison system is only part of the penal system, and the two must be considered together. Much harm has been caused in the past by attempts to tinker with the prison system without reference to general penal arrangements. The process by which a man gets sent to prison is quite as important as what is done to him when he gets there.

Every form of society has always contained those who offended against its laws; and from very early times society has made use of prisons. In the first instance these have usually been merely places of safe custody, pending such time as the inmates could be brought to trial, and so to judgment and the execution thereof. Micaiah, the son of Imlah, was remitted to prison until such time as Ahab had returned victorious from his campaign and had leisure to deal suitably with him. Incidentally, we may notice that he was ordered to be fed, while awaiting trial, with " the bread of affliction and the water of affliction." And we shall have something to say later on the permanence of this method of prison treatment. Prisons were also used for the more or less permanent detention of political offenders, and of what we should now call conscientious objectors. For example, John Bunyan was imprisoned for twelve years in Bedford Gaol because he persisted in preaching contrary to the provisions of the Act of Uniformity. But the idea of making a definite term of imprisonment the penalty for some offence against the laws of the State is a device of comparatively recent date. When the death penalty was no longer inflicted for all kinds of offences and when public castigations and mutilations began to get out of fashion, some new form of penalty had to be devised. And from this necessity has arisen our present prison system.

But the English system is peculiar, inasmuch as it has grown up from a dual origin. Transportation gave place to the convict prisons, and these have always been under the direct charge of the central Government, and were, until 1878, quite distinct from the "local" prisons. No correct estimation of the prison system is possible without a comprehension of this duality. Mr. and Mrs. Webb have fully recognised this. And so, although their work deals with the local government of prisons, they have included chapters on the convict prisons, and the system of penal servitude carried out therein.

The local prisons in this country sprang from two roots. There was the common gaol in a county under the nominal charge of the high sheriff, in a borough under the charge of the corporation. These were, originally, places of detention only. They were run for profit by the gaoler. The appalling character of these places has been described by John Howard, whose words are quoted by Mr. and Mrs. Webb. The conditions under which they were worked sound almost incredible to-day. Not only did an inmate's treatment depend entirely upon the remuneration which he was able to give to the gaoler, but there were fees to be paid not only on arrival, but also on discharge. So it actually happened that a man could be found not guilty at his trial and yet be taken back to gaol because he was unable to pay the discharge fees. There was also the house of correction or bridewell. This was, originally, a department of the machinery of the Poor Law, and was intended for what we should now call able-bodied paupers or vagrants. By the end of the seventeenth century they had, however, become places of punishment for minor offences. These two classes of prison gradually merged into one. But they were only made identical by statute in 1865. The filthy, insanitary, and degrading conditions existing in these places can be found described in Howard's famous book. And we need hardly say that medical attention in them was practically non-existent. As they were run for profit, the first object of the gaoler was to avoid incurring expense. It was cheaper to chain inmates to the walls than to incur the cost of officers. And when Howard wished his own county to place the gaol on a proper footing, and to pay the gaoler a salary, the first objection taken by the county justices was that of the expense involved. This dread of expense, the "Geddes" feeling of the time, has recurred again and again in prison history.

Transportation to the American "plantations" had been in use since the days of James I., and was regularised as a part of the criminal law in 1717. But the Declaration of Independence by the United States brought this system to an abrupt end. And in 1778 an Act was passed for the establishment of "penitentiaries." But, soon after this, transportation to Australia was introduced, the "first fleet" going there in 1787. The history of transportation is of great interest, but we should wander from our present subject if we entered into this.

In 1792 Jeremy Bentham proposed to erect a "model prison." He laid most stress upon the reformatory side of imprisonment. He proposed to effect reformation by means of religious instruction, combined with associated but silent labour. The place was, in fact, intended to be a kind of Borstal institution. He was so confident of success that he offered to be mulcted in a money fine for each one of his prisoners who was reconvicted after release. He proposed to raise money by making his institution a kind of public show, and he also thought that this would provide unofficial inspection. Nothing, however, was done in this direction until the building of Millbank was begun in 1812. We may note that Millbank was one of the most expensive buildings ever erected in the world, costing, from first to last, not less than three-quarters of a million of money.

In 1791 there was passed what we may call the first "Prisons Act." Unfortunately, most of its provisions were only permissive. And it was ignored by neglectful counties, just as the Mental Deficiency Act of 1913 has been ignored even in our own day.

"Hulks" were also established, first as a substitute for, and then as a preliminary stage of transportation. And the prisoners confined in these vessels worked in the national dockyards.

Pentonville Prison was built in 1842, as a "model prison," and served for many years as a preliminary place of confinement, first for transportees, and then for penal servitude prisoners. And the plan of wings radiating from a centre, introduced at Pentonville, as also at Millbank, has served as a model for many of our local prisons. This type of building undoubtedly has advantages from the point of view of ease of administration. But there are obvious drawbacks from the hygienic aspect, as also from the still more important aspect of the classification of prisoners.

For many years great controversy was waged around certain points of prison administration, *e.g.*, the advantages of separate confinement, the question of dietary, and the problem of prison labour. It must be kept in mind that the objects aimed at were, and still are, very complex. The original idea of a prison had simply been that of a place of safe custody. But to this had been added, as an essential, the maintenance of the prisoners in health, so far as the standard of the time went. It was also assumed that imprisonment must be made deterrent, both to the prisoner himself and to others. The desirability of introducing a reformatory element had also begun to be recognised. And, lastly, there was the motive of economy. These aims are quite distinct; they are, to some extent, mutually conflicting; and the grave differences of opinion which exist as to the relative importance which should be attached to these aims has caused the controversy to continue to the present day.

Solitary confinement had been introduced in America, probably as a reaction against the admitted evils of promiscuous and unrestrained association. It had its advocates in this country, and it was given a trial here. But the results were so appallingly bad that it was soon given up. And the real controversy raged over the question of separate confinement, and the system in which a prisoner worked in his cell all day, but was, at any rate in theory, constantly visited by prison officials. This was the plan adopted in the preliminary stages of penal servitude, and was, until fairly recent date, the system under which all local prisoners served their sentences.

The question of prison dietary is a complicated one. A great difficulty is that all meals have to be served in the cells. (This rule does not apply to prisoners associated in hospital or elsewhere, on medical grounds.) As a consequence, a weighed or measured quantity of food has to be allotted to every prisoner at each meal. The inevitable result is waste. Under the original local management there was almost every possible variety of diet, ranging upwards from a mere subsistence allowance. Constant attempts were made by the Home Office to secure the adoption of a uniform dietary scale. In 1864 a commission laid down the principle that the diet was not to be used as a penal instrument. And this has been, theoretically, the governing principle ever since. It is hard, however, to reconcile this principle with the system of dietary punishment, which has for years been the main form of punishment for offences against prison discipline. It is of course true that this, or or any other form of prison punishment, is never inflicted without the approval of the medical officer. But this method of punishment is really an anachronism and should be abolished. The question may be asked : What, then, is to be done with a prisoner who is guilty of breaches of prison rules ? The answer is that, when punishment is considered necessary, it should be awarded by means of the forfeiture of privileges. This method is in use in asylums. It has been constantly urged that a prison dietary should not be superior to that of the "lowest class of free-men outside prison." Even if this standard could be definitely ascertained, its adoption would lead to peculiar consequences. And the true principle is that laid down by the committee of 1864, that "the quality and amount of the dietary ought to be determined, not by the standard of any class of labourers, but by the actual necessities arising out of the prisoners' circumstances," and that, in this respect, the duties of the authorities are strictly analogous to that which they perform in other matters which involve the prisoners' health. A uniform diet was prescribed for all prisons when the local prisons were taken over in 1878. This scale has been altered on several occasions. Its application is, in all cases, subject to the discretionary powers of the medical officer. And this arrangement seems to be the best which can be devised, some scale of dietary being necessary.

The problem of prison labour was solved in various ways. Some local authorities attempted to make their institutions less costly by running what were, practically, factories, this system being brought to its greatest perfection at Wakefield. But the general idea was that prison labour should be as hard, as uninteresting, and as degrading as possible, and particularly that it should be unproductive. This last requirement was attained by the introduction of the "crank" and the "treadwheel." The former was definitely unproductive, and has long been discarded. The latter was not given up until 1902. In the latter years of its existence it had been made into a productive form of labour by the utilisation of the power for industrial purposes. The problem has been further complicated by the strong objection of the trade unions to any competition, from prison sources, in the ordinary market. Whether this objection is well founded is not for us to say. Nor need we discuss the question as to whether the present compromise, by which prison employment is limited to the manufacture of goods for Government departments, is really a reasonable one. Suffice it to say that if prison labour is to be used as a means of moral regeneration, it must be as varied, as instructive, and, above all, as conducive to self-expression, as possible. There are, of course, very great difficulties in providing employment for the large number of quite unskilled persons who are sentenced to short terms, and who form a considerable proportion of our prison population. The battle between the local authorities and the Home Office went on, with varying fortunes, for many years, the Home Office, by means of its inspectors, getting more and more power into its hands. Finally, by the Act of 1877, the central Government took over all the then existing local prisons, 113 in number, and the history of prisons under local government came to an end.

But meanwhile another system had been growing up which has had an immense effect upon our prison system. What is known as "penal servitude" was invented, at first as an alternative to, and later as a substitute for, transportation. Large "public works" prisons, as they were called, were built in various parts of the south of England, the inmates of which were to be employed on great works of public utility. Prisons of this kind existed at Portland, Portsmouth, Chatham and elsewhere. Of these, only Dartmoor and Parkhurst (the invalid convict prison) now exist in their original form. Portland and Aylesbury are now used as Borstal institutions. The buildings at Dover still stand on the cliff, but the prison has long been closed. The other buildings have disappeared. But the great breakwater at Portland and the dockyard basins at Chatham still remain as perpetual memorials of the convicts' labours.

The management of the convict prisons had always tended to be of a "military" character. And when in 1878 the two prison services were made one the then directors of convict prisons became also the Prison Commissioners and took over the rule of the local prisons. As a natural result, the tendency has been to accentuate the military element in the management of the local prisons. The effects of this have been bad, in various ways. There was, for many years, a marked tendency to the appointment of retired military officers as governors of prisons. This was, in part, due to a desire to provide posts for men whose previous training unfitted them for ordinary employments. But it was, also, due to the idea that military men were most competent to administer "discipline." Now discipline, in the sense of a graduated hierarchy of authority, we must always have, in some form, in any institution, unless complete anarchy is to prevail therein. But military discipline is not only objectionable in itself, but is utterly unsuited to proper prison conditions. Military discipline deals with men in the mass. It aims at making a number of men act as one, and thus it directly tends to discourage individuality. Now this is the very last thing which should be attempted with prisoners. For the primary necessity for any scientific dealing with offenders is to promote the growth of individuality and of self-expression. Again, military methods always tend to the production of a stereotyped uniformity, and the worship of this particular idol has been the bane of the prison service. Yet again, military men are always prone to confuse discipline with its outward expression. Real discipline is by no means the same thing as outward signs of respect for those of superior rank. And lastly, the military mind has a strong aversion from and jealousy of science. As a result, the medical side of the prison service, just as was the medical department of the army, was slighted and thwarted. And this defect has only begun to be remedied of quite recent years.

The amalgamation of the local and convict services produced another curious result. The convict service, as a department of Government, was the older, and its rulers, as we have just explained, absorbed the local service. As a consequence of this, the convict service regarded itself as superior. For many years the subordinate ranks of the two services were quite distinct, and the warder's pay in the convict service was higher than that of the corresponding grade in the sister service. Had this idea of superiority been confined to the officials, nothing worse than a little harmless vanity would have resulted. But the feeling spread to the inmates. And there is good evidence that convicts tended to regard themselves as something better than mere local prisoners. This was an unfortunate feeling to produce in what was, in theory, the worse type of offenders. And the whole régime of the convict prisons tended to increase this feeling of superiority. The routine was more dignified and more imposing. And not only was the convict made more of, but he was, in various respects, actually better off than a local prisoner. For example, it was possible for him to earn remission of sentence, whereas it was not until 1907 that this boon was conferred upon many local prisoners. The possible remission which can be earned by a male convict is, even now, a larger proportion of his sentence than can be earned by a local prisoner, and in the case of a female convict it is much larger. The convict and the local services have been, for some years, growing more alike. The union of all ranks of officers is now complete. It is a grave question whether it is now advisable to continue the existence of two forms of imprisonment.

Since the amalgamation of 1878 there has been no essential change in the prison service. Alterations in detail there have been. But the main alterations have been brought about by the introduction of legislation which has produced changes, not in the prison but in the penal system. Some of these developments we shall discuss in our next number.

The question may be asked, was the amalgamation a good thing. It was, probably, inevitable, although the main immediate reason for making it was to relieve the county authorities of expense. Few would now wish to return to the old arrangement. Large localities might manage their prisons well. But some of the country districts would require constant keeping up to date, as is now the case with some other public services. The system of central government has, however, very grave disadvantages. And some of these, with suggestions as to their possible remedies, we shall discuss next month.

